# Suppression of miR-16 promotes tumor growth and metastasis through reversely regulating YAP1 in human cholangiocarcinoma

**DOI:** 10.18632/oncotarget.17832

**Published:** 2017-05-12

**Authors:** Sheng Han, Dong Wang, Guohua Tang, Xinxiang Yang, Chenyu Jiao, Renjie Yang, Yaodong Zhang, Liqun Huo, Zicheng Shao, Zefa Lu, Jiawei Zhang, Xiangcheng Li

**Affiliations:** ^1^ Liver Transplantation Center of The First Affiliated Hospital, Nanjing Medical University, Nanjing, Jiangsu Province, P.R. China

**Keywords:** CCA, miRNA, proliferation, metastasis, prognosis

## Abstract

**Background & Aims:**

Aberrant expression of microRNAs is associated with many cancers progression. Many studies have shown that miR-16 is down-regulated in many cancers. However, its role in cholangiocarcinoma (CCA) is unknown.

**Methods:**

Quantitative real-time PCR (qRT-PCR) was developed to measure miR-16 expression in CCA tissues and cell lines. CCK-8, colony formation and transwell assays were used to reveal the role of miR-16 in CCA cell proliferation and malignant transformation *in vitro*. The loss-and-gain function was further validated by subcutaneous xenotransplantation and tail vein injection xenotransplantation model *in vivo*. Dual-luciferase reporter assay was performed to validate the relationship of miR-16 with YAP1.

**Results:**

MiR-16 was notably downregulated in CCA tissues, which was associated with tumor size, metastasis, and TNM stage. Both *in vitro* and *in vivo* studies demonstrated that miR-16 could suppress proliferation, invasion and metastasis throughout the progression of CCA. We further identified YAP1 as a direct target gene of miR-16 and found that miR-16 could regulate CCA cell growth and invasion in a YAP1-dependent manner. In addition, YAP1 was markedly upregulated in CCA tissues, which was reversely correlated with miR-16 level in tissue samples. Besides, Down-regulation of miR-16 was remarkably associated with tumor progression and poor survival in CCA patients through a Kaplan–Meier survival analysis.

**Conclusions:**

miR-16, as a novel tumor suppressor in CCA through directly targeting YAP1, might be a promising therapeutic target or prognosis biomarker for CCA.

## INTRODUCTION

Cholangiocarcinoma (CCA) is a highly aggressive tumor derived from bile duct epithelial cells. In recent decades, the incidence and mortality of CCA have been increasing globally [1, 2]. The survival rate and prognosis of patients with CCA is dismal on account of the early invasion and metastasis [3]. Although radical surgery is the only curative treatment for CCA, patients benefit little because they are usually diagnosed at an advanced stage [4]. Therefore, an improved understanding of the pathogenesis of CCA to find novel diagnostic and therapeutic approaches are urgently needed [5, 6].

MicroRNAs (miRNAs), as one member of small non-coding RNAs family, have been confirmed by abundant evidences to affect malignancies with binding to the partial complementary recognition sequences in the 3´-untranslated region (3´-UTR) of the mRNA, which results in target mRNA translation inhibition or degradation [7, 8]. Lots of miRNAs play a pivotal role in tumorigenesis such as cell proliferation, migration, invasion, apoptosis, and metastasis [9–12]. As is known to all, the tumor suppressor miR-16 is observably decreased and its function has been studied all the time in different cancers, including lung cancer, osteosarcoma, breast cancer, glioma, laryngeal carcinoma and so on [13–15]. However, to this end, we have poor understanding of the detailed role of miR-16 in cholangiocarcinoma.

Yes-associated protein 1 (YAP1), as a direct downstream effector of the tumor suppressive hippo pathway, has been found to be elevated in various cancers, such as liver cancer [16], colorectal cancer [17], ovarian cancer [18], gastric cancer [19] and non–small-cell lung cancer [20]. However, the regulatory mechanism of YAP1 expression in CCA remains largely unknown.

In the present study, we verified the significant downregulation of miR-16 and upregulation of YAP1 in CCA cell lines and tissues. In addition, miR-16 overexpression dramatically suppressed CCA cell proliferation, growth and migration through inhibiting YAP1 expression. Furthermore, we explored the potential mechanism of miR-16 and found that YAP1 was a direct target of miR-16 in CCA. All these findings might help us to find new diagnosis methods and therapeutic strategies for the treatment of CCA.

## RESULTS

### MiR-16 is significantly down-regulated in CCA tissues and cell lines

**Figure 1:**
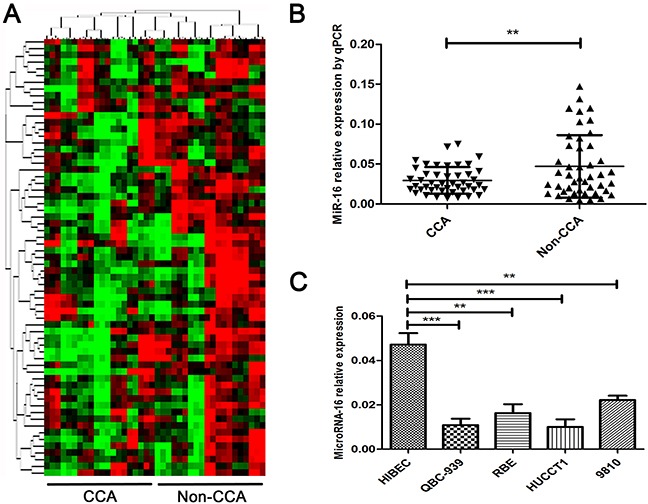
MiR-16 is pronouncedly down-regulated in human CCA tissues and cell lines **(A)** MiRNA microarray detection in 20 paired CCA corresponding adjacent tumor tissues and tumor tissues. Data was presented as heat-map. Red indicated the overexpression miRNAs while green indicated the down-regulated miRNAs. **(B)** Real-time PCR was performed to measure the expression level of miR-16 in 45 pairs of CCA and the corresponding non-CCA tissues, where U6 was used as an endogenous control. **(C)** The expression levels of miR-16 was determined by real-time PCR in 4 CCA cell lines as well as the normal biliary epithelial cell line HIBEC, with U6 as an internal control. All data were from at least three independent experiments. Data are presented as means ± SEM and analyzed with Student's t test (**P < 0.01, ***P < 0.001).

The miRNA high-throughput microarray was applied to screen the potential miRNAs involved in the pathogenesis of CCA. 20 paired samples were employed in the screening. As presented in Figure 1A, we found an aberrant different expression level of miRNAs in the tumor tissues and corresponding adjacent tissues. The top 11 miRNAs with the highest different were considered as candidates (Supplementary Table 1). We further validated the expression of the 11 candidates in a larger CCA sample size. As indicated in Figure 1B, miR-16 was pronouncedly downregulated in 45 CCA tissues when compared to their corresponding non-CCA tissues. Analogously, miR-16 expression level was also down-regulated in 4 widely used CCA cell lines (HUCCT1, QBC939, RBE, 9810) relative to normal biliary epithelial cell line (HIBEC) (Figure 1C). Collectively, these results suggested that miR-16 expression is down-regulated in human CCA tissues and cell lines.

### MiR-16 inhibits CCA cell proliferation and invasion *in vitro*

**Figure 2:**
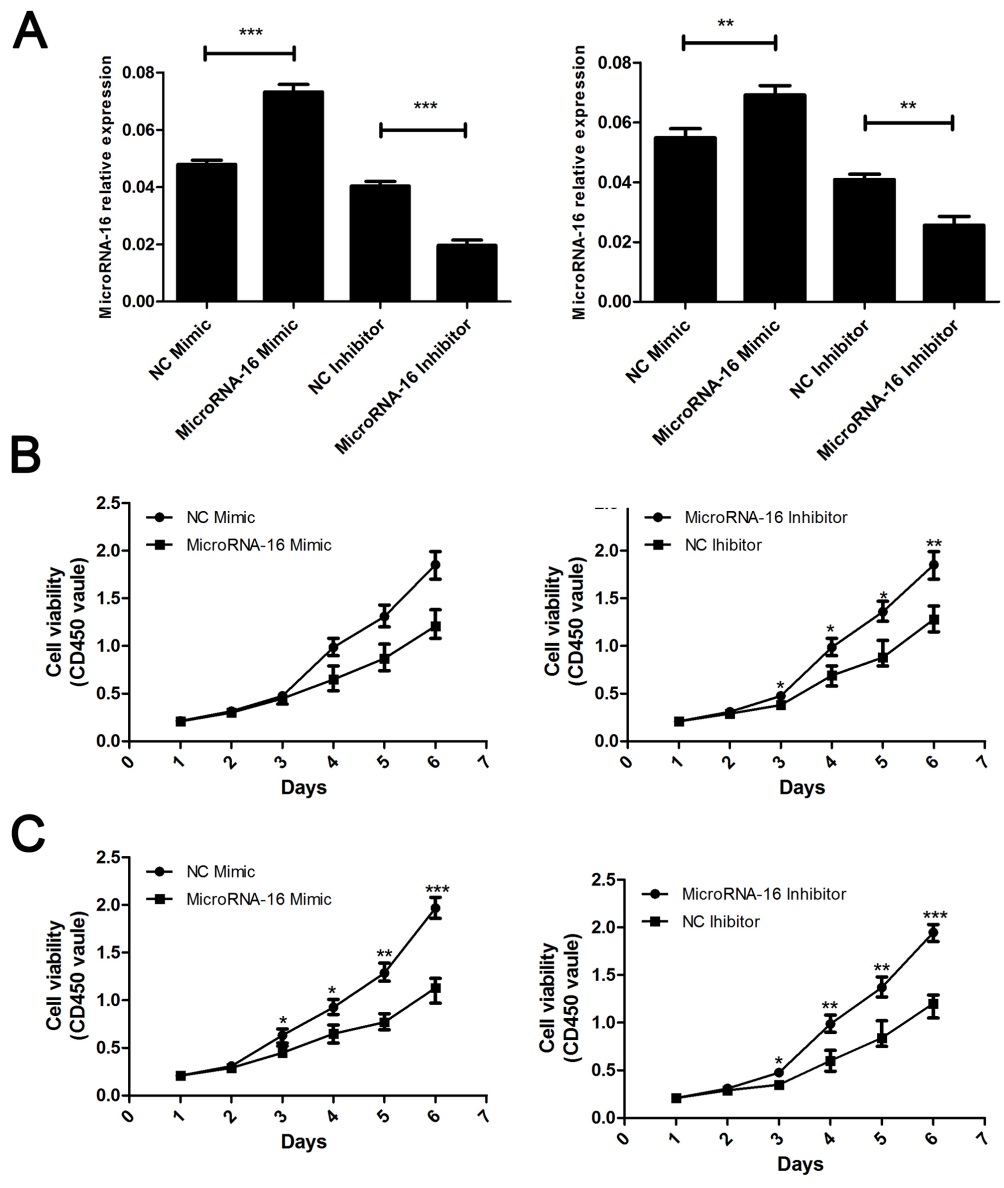
MiR-16 suppresses CCA cell proliferation *in vitro* by CCK8 assay **(A)** Real-time RT-PCR was conducted to examine the expression levels of miR-16 in CCA cells transfected with miR-16 mimic or inhibitor, respectively. The measure unit in Y axis is 2-ΔCt value. The left panel indicated the HUCCT-1 while the right panel indicated the QBC939 cell line. **(B and C)** CCK8 assay were employed to detecting the proliferation of CCA cells. The upper panel indicated the HUCCT-1 while the lower panel indicated the QBC939 cell line. CD450 stand for the wave length. The absorbance value at a wave length of 450nm was used as an indicator of cell viability. Data are presented as means ± SEM and analyzed with Student t test (**P < 0.01, ***P < 0.001).

**Figure 3:**
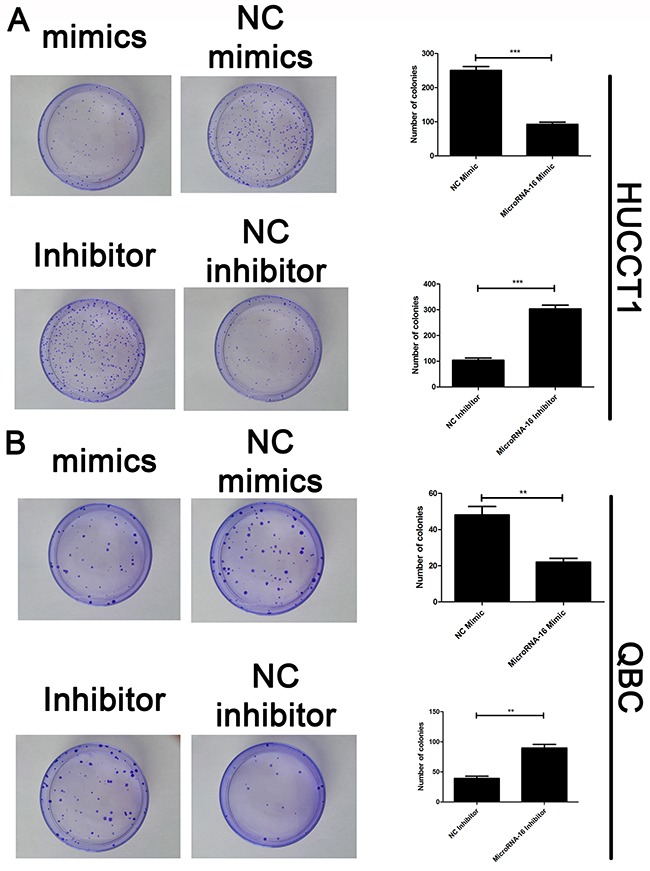
MiR-16 suppresses CCA cell proliferation *in vitro* by colony formation assay Colony formation assay was employed to detecting the proliferation of CCA cells. **(A)** HUCCT1 cell line; **(B)** QBC cell lines. Data are presented as means ± SEM and analyzed with Student's t test (**P < 0.01, ***P < 0.001).

**Figure 4:**
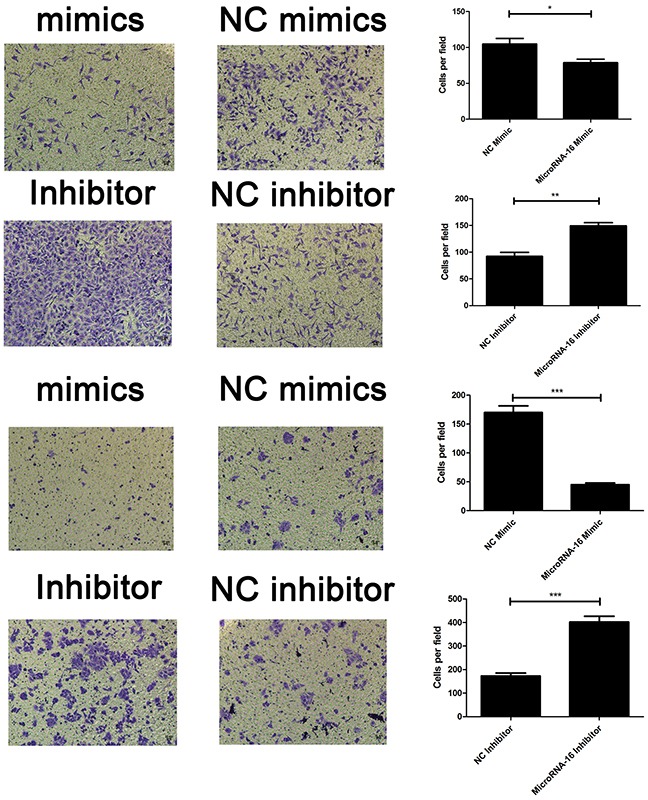
MiR-16 inhibits CCA cell invasion *in vitro* Transwell assay was conducted to assess the effect of miR-16 overexpression or knockdown on CCA cell invasion capacity. The number ofcells that invaded through the membrane was counted under a light microscope with ×100 magnification. The upper panel indicated the HUCCT-1 while the lower panel indicated the QBC939 cell line. *P<0.05; **, P < 0.01; ***, P < 0.001.

To reveal the role of miR-16 in CCA, miR-16 level in HUCCT-1 and QBC939 cells transfected with miR-16 mimic or inhibitor, was examined using qRT-PCR. As showed in Figure 2A, cells treated with miR-16 mimic showed a significant increase in the miR-16 level, while transfection of miR-16 inhibitor notably inhibited the miR-16 level, compared to the negative control group. Overexpression of miR-16 remarkably suppressed CCA cell proliferation, while knockdown of miR-16 significantly enhanced CCA cell proliferation as determined by the CCK-8 (Figure 2B and 2C) and colony formation (Figure 3A and 3B). Cell apoptosis was detected upon miR-16 gain- and loss-expression using flow cytometry. The results showed that overexpressing of miR-16 promoted HUCCT-1 cell apoptosis from 6.81% to 12.11%, and knockdown of miR-16 decreased cell apoptosis from 9.64% to 3.41% (Supplementary Figure 1A and 1B). Similar results were also observed in QBC939 cell line (Supplementary Figure 1C and 1D). In order to investigate whether miR-16 is associated with CCA cell invasion, transwell assay was performed. Results revealed that upregulation of miR-16 significantly inhibited the invasion capacity and silencing miR-16 expression notably increased the invasion abilities in both HUCCT-1 and QBC-939 cells (Figure 4).

### YAP1 is a direct target of miR-16 in CCA cells

**Figure 5:**
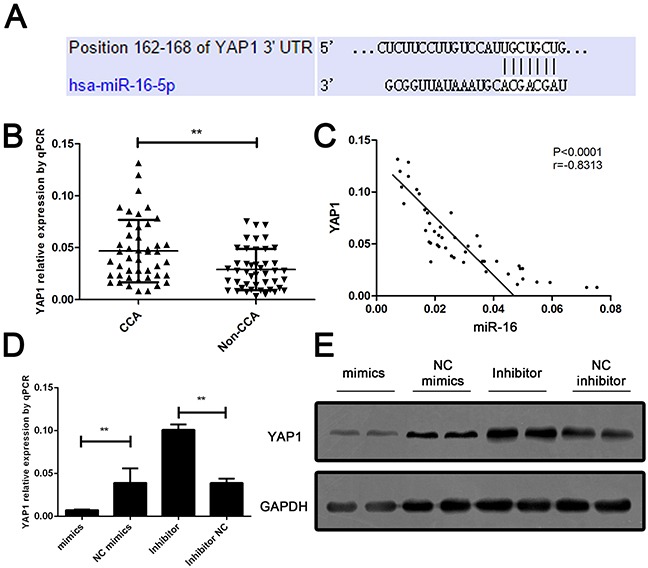
YAP1 is a down-stream target and negatively regulated by miR-16 in CCA cells **(A)** The sequence alignment of human miR-16 with 3′ UTR of YAP1. **(B)** Real-time PCR was performed to measure the expression level of YAP1 in CCA and the corresponding non-CCA tissues, where GAPDH was used as an endogenous control. P value was calculated by Student's t test. **(C)** Pearson correlation analysis was employed to calculate the correlation between the expression of miR-16 and YAP1. **(D)** The mRNA expression of YAP1 was detected in cells treated with either miR-16 mimics or inhibitor. Protein expression of YAP1 was detected in cells treated with either miR-16 mimics or inhibitor. Data are presented as means ± SEM and analyzed with Student t test (**P < 0.01).

To further assess the mechanism of miR-16-induced CCA cell growth and invasion suppression, bioinformatic analysis was performed to identify the potential down-stream target of miR-16. The candidate targets of miR-16 were predicted in multiple databases including miRbase, Targetscan, Pictar and miRNA target. As listed in Supplementary Table 2, six candidates were enrolled according to the score predicted by database. We further detected the expression of six candidates in CCA patients; YAP1 was confimed as the most significant upregulatd target genes in the tumor tissues of CCA patients (Figure 5A and 5B). Further Pearson analysis revealed that YAP1 was reversely correlated with the miR-16 level in CCA tissues (Figure 5C). Moreover, the effect of miR-16 on YAP1 expression was examined in CCA cells. Result showed that YAP1 expression was markedly decreased by overexpressing of miR-16 and significantly increased by down-regulation of miR-16 (Figure 5D and 5E).

**Figure 6:**
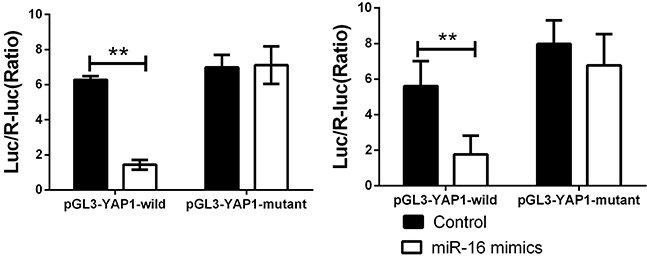
miR-16a could directly binding with YAP1 3′UTR Cells were co-transfected with miR-16 mimics or Control, Renilla luciferase vector pGL3-SV40 and YAP1 3′UTR luciferase reporters for 48h. Both firefly and Renilla luciferase activities were measured in the same sample. Firefly luciferase signals were normalized with Renilla luciferase signals. ** indicated remarkable significant difference (P<0.01). The left panel indicated the HUCCT-1 while the right panel indicated the QBC939 cell line. All tests were performed in triplicate and presented as means ± SEM.

To further confirm this bioinformatic predication, we constructed wild type and mutant type of YAP1 3′-UTR and conducted luciferase reporter assay. It can be seen that the luciferase activity was significantly decreased in CCA cells co-transfected with miR-16 mimic and wild type of YAP1 3′UTR. However, it was unchanged in other groups compared to the control group (Figure 6). These findings demonstrated that YAP1 is a target gene of miR-16 in CCA cells, indicating that miR-16 negatively regulates YAP1 expression in CCA cells via directly binding to the 3′-UTR of its mRNA.

### MiR-16 regulates CCA cell growth and metastasis in a YAP1- dependent manner

**Figure 7:**
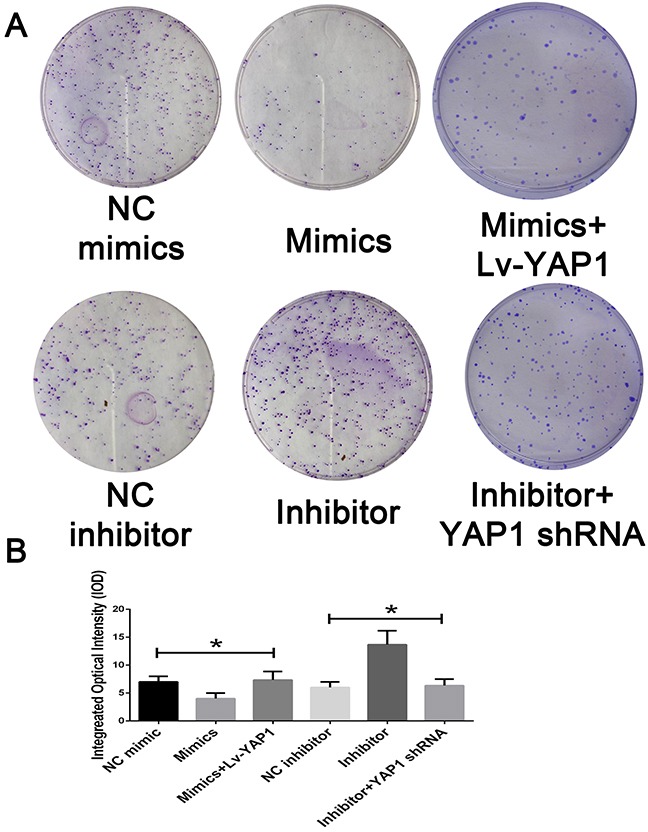
Mir-16 inhibits cell proliferation through the YAP1-dependent manner **(A)** The colony formation assay was employed to detecting the proliferation of CCA cells. **(B)** The integral optical density value was measured and analyzed. * indicated remarkable significant difference (P<0.05). All tests were performed in triplicate and presented as means ± SEM.

**Figure 8:**
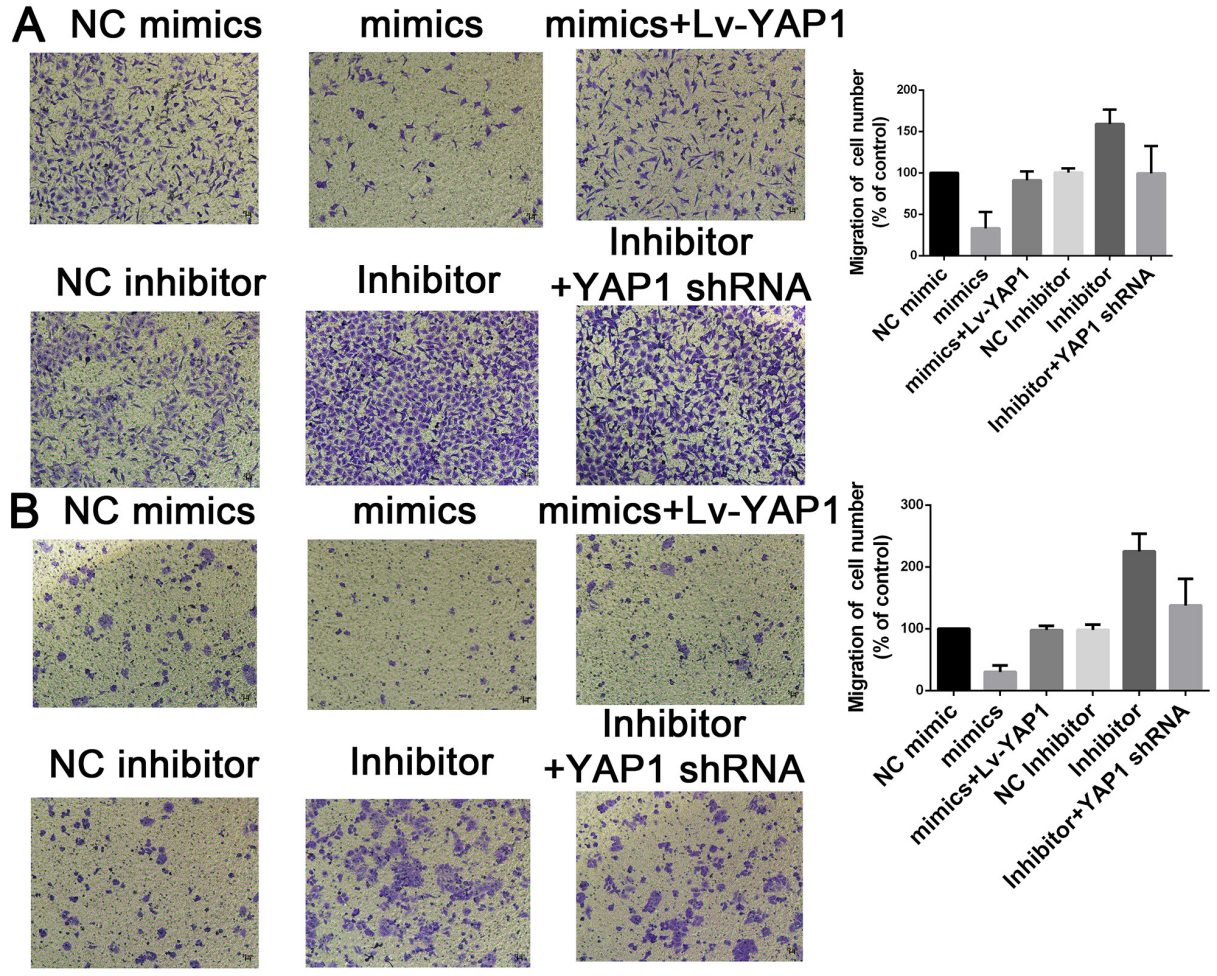
Mir-16 inhibits cell invasion through the YAP1-dependent manner Transwell assay was conducted to assess the effect of miR-16 overexpression or knockdown on CCA cell invasion capacity. The number ofcells that invaded through the membrane was counted under a light microscope with ×100 magnification. **(A)** HUCCT1 cell line; **(B)** QBC cell lines.

Based on the above data, we speculated that YAP1 might be involved in miR-16-mediated CCA cell growth and invasion. To confirm our speculation, we assessed the colony formation and transwell assay when miR-16-overexpressing cells were further transfected with YAP1-overexpressing lentivirus. Interestingly, YAP1 overexpression dramatically rescued the suppressing effects of miR-16 up-regulation on CCA cell proliferation. On the contrary, RNAi against YAP1 almost completely abolished the promoting role of miR-16 down-regulation on CCA cell proliferation (Figure 7). Additionally, YAP1 overexpression significantly antagonized the inhibitory effects of miR-16 up-regulation on CCA invasion while YAP1 down-regulation obviously reversed miR-16 knockdown-induced invasion potential of CCA cells (Figure 8).

**Figure 9:**
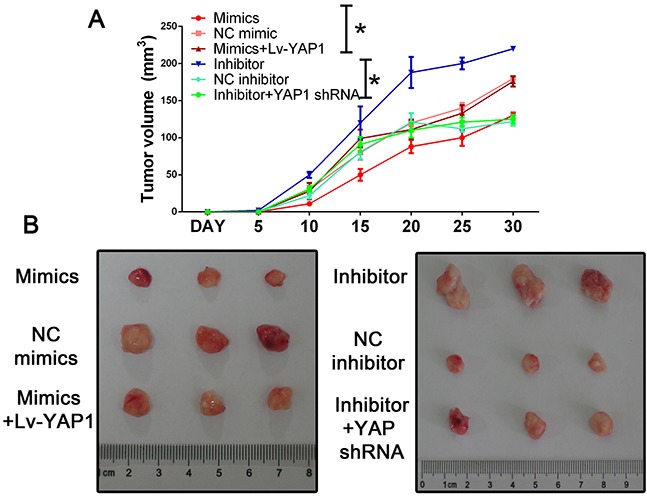
Mir-16 inhibits tumor growth through the YAP1-dependent manner **(A)** Nude mice were subcutaneously transplanted with cells stably expressed with miR-16 mimics/inhibitor or control (n=5). The weight and volume of each tumor was calculated every 5 days. Data was presented with mean ± SEM. Student's t test was used to evaluate the statistical significance of these experiments, as compared to the control. The left panel indicated the HUCCT-1 while the right panel indicated the QBC939 cell line. * indicated P < 0.05. **(B)** The representative tumor in each group.

**Figure 10:**
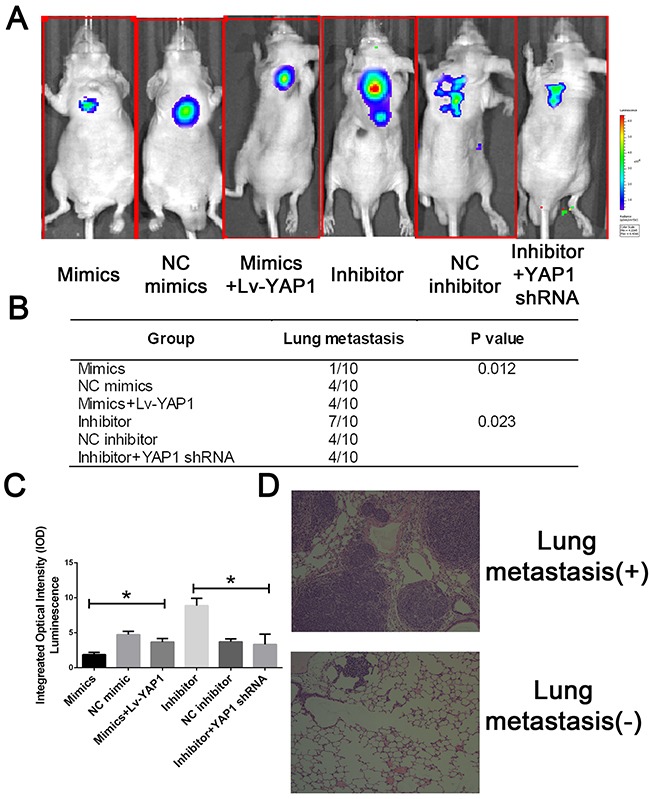
MiR-16 inhibits CCA metastasis *in vivo* through the YAP1-dependent manner **(A)** The lung metastatic nude was detected by IVIS@ Lumina II system. **(B)** The lung metastatic nude was recorded was analyzed in each group. **(C)** The integral optical density value of metastatic nude was calculated. **(D)** The representative H-E stain of lung tissues with or without metastasis.

To further evaluate the effects of miR-16 *in vivo*, we established a xenograft model in nude mice in which QBC939 cells were utilized. Compared with the control group, miR-16 overexpressed mice resulted in a significant reduce of tumor weight and tumor volume while miR-16 inhibited mice presented tumors with increased weight and size (Figure 9). However, the promotion of tumor growth could be abolished by either co-treated cells with miR-16 mimics and YAP1 overexpression lentivirus or co-treated with miR-16 inhibitor and YAP1 shRNA lentivirus, indicating the function induced by miR-16 might be in a YAP1- dependent behavior. We also validated the gain-and-loss function of miR-16 in tumor metastasis. QBC939-miR-16 mimic, QBC939-miR-16 inhibitor, and their control cells were injected into nude mice via the tail vein respectively. MiR-16 overexpression in QBC939 cells resulted in observable decrease of the number of mice with lung metastasisand fewer metastatic nodules in the pulmonary tissues of each mouse, compared with their control group whereas miR-16 down-regulation led to the promotion results (Figure 10). The detailed information of metastasizing nude has been listed in Figure 10B and 10C. We further investigated the metastasizing nude by H-E stain as presented in Figure 10D. These observations provided evidence that miR-16 is a potent inhibitor of CCA metastasis.

In summary, we infer that YAP1 is involved in miR-16-mediated CCA cell growth and metastasis and up-regulation of YAP1 may due to the down-regulation of miR-16 in CCA.

### Down-regulation of miR-16 is remarkably associated with tumor progression and poor survival in CCA patients

**Figure 11:**
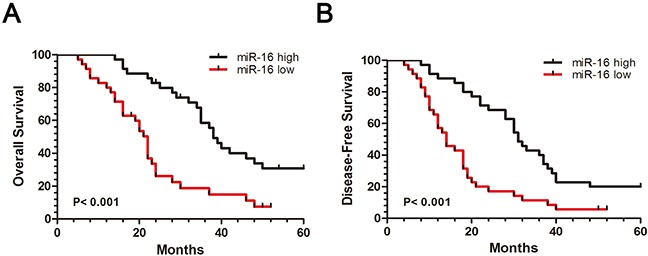
Down-regulation of miR-16 is associated with CCA poor prognosis **(A)** Patients with high miR-16 expression had a better disease-free survival (DFS) than patients with low miR-16 expression. **(B)** Patients with high miR-16 expression had a better overall survival (OS) than patients with low miR-16 expression. 45 CCA samples were divided into miR-16 high expression group (n=35) and low expression group (n=35), while median was used as cut off.

To further confirm the expression significance of miR-16 in CCA, we evaluated the correlation between clinicopathological characteristics of these CCA patients and miR-16 expression. All these CCA samples were divided into miR-16 high expression group (n=22) and low expression group (n=23), median was used as cut-off value. Statistical results showed that miR-16 expression was remarkably associated with tumor size (P=0.004), metastasis (P=0.002), and AJCC TNM stage (P < 0.001) in CCA patients, suggesting that down-regulation of miR-16 may contribute to the malignant progression of CCA (Table 1). In addition, to analyze the prognostic significance of miR-16 expression, Kaplan–Meier survival curve was carried out in a set of 45 CCA patients with integral follow-up data. It can be seen that patients with low expression of miR-16 exhibited a worse disease-free survival (DFS) than those with high expression of miR-16 (Figure 11A). Likewise, a statistically significant association between low miR-16 expression and short overall survival (OS) was also demonstrated in CCA patients (Figure 11B). Taken together, these results implied that down-regulation of miR-16 can predict poor survival of CCA.

**Table 1:** The clinicopathological relevance analysis of miR-16 expression in cholangiocarcinoma patients

Characteristic	miR-16			YAP1		
	Low	High	P value	Low	High	P value
**All cases**	23	22		22	23	
**Age**			0.884			0.884
<60	11	11		11	11	
≥60	12	11		11	12	
**Gender**			0.652			0.181
Male	12	10		13	9	
Female	11	12		9	14	
**Differentiation grade**			0.891			0.336
Well	11	12		13	10	
Moderate	8	7		5	10	
Poorly	4	3		4	3	
**Tumor Size(cm)**			**0.004**			**<0.001**
≤5cm	11	20		20	3	
>5cm	12	2		2	20	
**Tumor Origination**			0.906			0.985
Left	13	11		12	12	
Right	9	10		9	10	
Bilateral	1	1		1	1	
**TNM stage**			**<0.001**			**<0.001**
I-II	8	19		20	7	
III	15	3		2	16	
**Metastasis**			**0.002**			**0.002**
Yes	18	7		7	18	
No	5	15		15	5	

## DISCUSSION

In the present study, we revealed that miR-16 expression was significantly down-regulated in CCA tissues compared with the corresponding non-CCA tissues. Besides, decreased expression of miR-16 was also found across a panel of CCA cells compared to normal bile duct epithelial cell. Moreover, CCA tissues with large tumor size, metastasis, or advanced TNM stage showed a significant down-regulation of miR-16 expression, which suggested that miR-16 might be involved in CCA carcinogenesis. Further, *in vitro* and *in vivo* experiments demonstrated that miR-16 could inhibit CCA cell proliferation, colony formation ability and decreased cell invasion. Moreover, YAP1, as a candidate human oncogene in multiple tumors, was identified as a down-stream target gene of miR-16 for the first time. Therefore, we speculated that miR-16 was capable of suppressing CCA cell proliferation and invasion by enhancing YAP1 expression. As expected, the inhibitory effect of miR-16 up-regulation on CCA cell growth and invasion was significantly rescued by the YAP1-overexpressing plasmid, although the recovery was not 100% efficient compared with control cells, while the promoting role of miR-16 down-regulation on CCA cells was almost completely abolished by YAP1 knockdown. Thus, our data indicated that miR-16 regulates CCA cell growth and invasion in a YAP1-dependent manner. Finally, we observed an obvious up-regulation of YAP1 in CCA tissues compared to their matched non-CCA tissues, as well as a negative correlation between YAP1 and miR-16 levels in the examined CCA tissues. Given that YAP1 is an oncogene in many cancers, we conclude that miR-16 inhibits the growth and invasion of CCA cells at least partially by targeting YAP1.

MiR-16 has been reported to play a suppressive role in different human cancers. For example, Ke et al. showed that miR-16 could inhibit growth and motility in non-small cell lung cancer cells by targeting hepatoma-derived growth factor (HDGF) [21]. He et al. found that miR-16 suppressed carcinogenesis and progression of nasopharyngeal carcinoma via targeting fibroblast growth factor 2(FGF2), inactivating the PI3K/AKT and MAPK signaling pathways [22]. In cutaneous T-cell and other non-Hodgkin lymphomas, miR-16 was suggested to mediate the regulation of a senescence-apoptosis switch and induce cellular senescence [23]. Moreover, Chatterjee also demonstrated that overexpression of miR-16 could sensitize paclitaxel resistant lung cancer cells to paclitaxel by inducing apoptosis via inhibiting anti-apoptotic protein Bcl-2 [13]. However, whether and how miR-16 is involved in CCA progression is still unknown. In the present study, we found that the miR-16 expression was remarkably decreased in CCA tissues and cell lines, and loss of miR-16 was tightly associated with the advanced malignancy of CCA that was involved in the progression of CCA. Moreover, we found that ectopic expression of miR-16 was markedly capable of preventing proliferation and invasion in CCA cells both *in vitro* and *in vivo*, while knockdown of miR-16 enhanced CCA cell growth and invasion, which suggested that miR-16 played a crucial role in cellular homeostasis that contributes to the development of CCA. Thus, our findings enriched the tumor suppressive role of miR-16 in CCA.

YAP1, a key downstream effector of Hippo tumor suppressor signaling pathway [24], regulates multiple cellular processes by activating several transcription factors, such as TEAD1-4 [25], and has been suggested to be a candidate human oncogene in multiple tumors [20, 26, 27]. Sun et al. demonstrated that YAP1 could promote the survival and self-renewal of breast tumor initiating cells by inhibiting Smad3 signaling pathway [28]. Wang et al. showed that YAP1 expression is involved in epithelial–mesenchymal transition in hepatocellular carcinoma [29]. YAP1 can also act synergistically to promote pancreatic cancer progression by hyperactivation of AKT signaling [30]. Notably, multiple miRNAs have been reported to regulate YAP1 expression in many cancers. Deng et al. delineated that miR-506 inhibits gastric cancer proliferation and invasion by directly targeting YAP1 [31]. Ling Xiao revealed that miR-138 may play a suppressive role in the growth and metastasis of NSCLC cells by targeting YAP1 [32]. Ruan T reported that miR-186 targets YAP1 to inhibit Hippo signaling and tumorigenesis in hepatocellular carcinoma [33]. In prostate cancer, miR-375 is involved in development of chemo-resistance to docetaxel through regulating YAP1 expression [34]. Tao J found that YAP1 activation is frequent in cHC-CCs and ICCs and correlates with SMAD family member 2 activation. Drug screening revealed that antiparasitic macrocyclic lactones inhibit YAP1 activation *in vitro* and *in vivo* [35]. In addition, genetic and chemical perturbation experiments demonstrate the requirement for Mediator and CDK9 in YAP-driven phenotypes of overgrowth and tumorigenesis [36]. However, whether YAP1 is directly regulated by microRNAs in CCA until now remains unclear.

To clarify the molecular mechanism underlying miR-16 induced CCA proliferation and metastasis, we used bioinformatics analysis to predict the potential targets of miR-16 and YAP1 was indicated as a theoretical miR-16 target. In turn, collectively data support that YAP1 acts as a direct down-stream target of miR-16 in regulating CCA cell growth and invasion. First, western blot analysis showed that miR-16 decreased expression levels of YAP1. Second, pathological data demonstrated that YAP1 expression was inversely correlated with miR-16 expression in clinical CCA samples. Third, the luciferase reporter assay with 3′UTR revealed miR-16 repressed YAP1 expression via interaction with YAP1-3′UTR elements. Finally, YAP1 expression markedly antagonized the function of miR-16 on CCA cell proliferation and invasion. Therefore, our results demonstrated a direct interaction between miR-16 and YAP1 and their critical role in CCA, which demonstrated that the targeting and inhibition of YAP1 by miR-16 may be a feasible therapeutic treatment for CCA.

In conclusion, we for the first time discovered that that miR-16, as a tumor suppressor in CCA via targeting YAP1, may serve as a promising therapeutic target for CCA.

## MATERIALS AND METHODS

### Human tissue specimens and cell culture

All CCA specimens were collected from CCA patients who underwent surgical resection from April 2008 to June 2011. The written informed consents were obtained for use of tissue samples prior to surgery, and the study was approved by the institutional ethics committee of Nanjing Medical University. Both the tumor and adjacent normal tissue were immediately stocked at liquid nitrogen after surgical removal and stored at −80°C until processed. The diagnosis of CCA was validated by two individual pathologists.

### Cell culture and transfection

Human CCA cell lines (HUCCT1, RBE, 9810, QBC-939) and human intrahepatic biliary epithelial cell lines (HiBEC) were cultured in Dulbecco's Modified Eagle's Medium (DMEM) with10% fetal bovine serum (FBS) and 1% penicillin-streptomycin in a humidified incubator containing 5% CO_2_ at 37°C.

MiR-16 mimic, inhibitor and respectively corresponding negative control were purchased from Gene Pharma (Shanghai, China). YAP1-overexpressing plasmid and YAP1-specific small interference RNAs (siRNAs) were also designed and chemically synthesized from Gene Pharma (Shanghai, China). Lipofectamine 2000 (Invitrogen, USA) was used for cell transfection according to the manufacturer's instructions.

### RNA extraction and quantitative real-time PCR

Total RNA was isolated and reverse transcribed to cDNA using the kit from TIANGEN Biotech Co. Ltd (Beijing, China). MiRNA cDNA Synthesis kit was used to reversely transcribe miRNA and EvaGreen miRNA qPCR Mastermix was used for quantification, which were purchased from ABM (Nanjing, China). U6 or Glyceraldehyde-3-phosphate dehydrogenase (GAPDH) was used as negative control to normalize the target gene expression. The primers were used for qRT-PCR as following: GAPDH, 5´-CTGGGCTACACTGAGCACC-3´ and 5´-AAGTGGTCGTTGAGGGCAATG-3´ and YAP1, 5´-TAGCCCTGCGTAGCCAGTTA-3´ and 5´-TCATGCT TAGTCCACTGTCTG.

### Immunohistochemistry (IHC)

After 4% paraformaldehyde fixation, the sections were deparaffinized. Antigens of the slides were retrieved by heating for 30 min in citrate buffer, pH 6.0. The slides were labeled with primary antibody in a blocking solution (1:100 dilution) at 4°C overnight, following by counterstained with hematoxylin. Light microscopy (Nikon, Tokyo, Japan) was used to acquire the images, and NIS-Elements v4.0 software was used to quantify the staining (Nikon).

### Cell proliferation and colony formation assay

Cell Counting Kit (CCK)-8 from Beyotime Institute of Biotechnology (Nanjing, Jiangsu, China) was used to measure cell proliferation. 2500 cells/well were seeded in 96-well plates. At 0, 24, 48 and 72 h, 200 μl fresh medium containing 10 μl CCK-8 was added into each well to replace the original medium. The spectrophotometer was used to measure the absorbance of each well (Thermo Scientific, Pittsburgh, PA, USA). For colony formation assay, after being seeded in 6-cm plate, cells were cultured for 7-12 days. After 4% paraformaldehyde fixation, cells were stained with 1% crystal violet for counting.

### Flow cytometric analysis of cell apoptosis

The apoptotic rate of cells was determined using Invitrogen Annexin V-fluorescein isothiocyanate (FITC) and propidium iodide (PI) double staining (Thermo Fisher Scientific, USA). Briefly, after 48 h of transfection with miR-16 mimics or inhibitors, the cells were harvested, washed twice with phosphate-buffered saline (PBS), resuspended in 100 μl Annexin-binding buffer and subsequently incubated with 5 μl Annexin V-FITC and 3 μl PI (50 μg/ml) for 30 min in the dark at room temperature. Early apoptotic cells were positive for Annexin V and negative for PI, and while late apoptotic cells were both Annexin V- and PI-positive. Apoptotic cells were recognized by a FACScan flow cytometer (Becton Dickinson, USA) and the data were analyzed using Cell Quest software (Beckman Coulter, USA).

### Western blotting

Total protein from cells or tumor tissues was extracted using a protein extraction kit (Applygen Technologies, China). Protein concentrations in different samples were measured using the BCA Protein Assay Kit (Pierce Chemical, USA). Proteins were resolved by sodium dodecyl sulfate-polyacrylamide gel electrophoresis and transferred to polyvinylidene difluoride membranes (Roche Diagnostics, USA), which were blocked in 4% dry milk at room temperature for 1 h. After incubation with the appropriate primary antibody overnight at 4°C, membranes were washed and incubated with the corresponding secondary antibodies included horseradish peroxidase (HRP) conjugated Goat anti-rabbit IgG for 2 h at room temperature. Then the protein bands of interest were visualized with the Odyssey system (LI-COR, USA). After washing, the protein bands of interest were visualized using the ECL Western Blotting Kit (Pierce Chemical, USA). The integrated density of protein bands was quantified using Image Lab software (Bio-Rad, USA).

### Cell invasion assay

Matrigel-coated invasion chamber (Becton Dickinson, USA) was used to analyze the invasion ability of CCA cells. A total of 5×104 cells were suspended in500μl DMEM without serum and added to the upper chamber, while 750μl DMEM containing 10 % FBS and10 μl/ml fibronectin (BD Biosciences, USA) was used as the nutritional attractant in the lower chamber. After 48 h of incubation, cells that invaded into the lower surface of the membrane were fixed in 4 % paraformaldehyde, stained with 0.5 % crystal violet, and counted with a microscope. At least six random microscopic fields (magnification, 100×) were analyzed for each insert.

### Luciferase reporter assay

The binding sites for miR-16 in the 3′UTR of human YAP1 was inserted into the pMIR-REPORT vector. Site-Directed Mutagenesis kit (Stratagene, Shanghai, China) was used to generate YAP1 3′UTR mutated versions, followed by sequencing confirmation. For luciferase reporter assay, after being seeded in 96-well plates, cells were transfected with wild-type or mutant luciferase reporter combined with miR-16 using lipofectamine 2000. Dual-Luciferase Reporter Assay System (E1910, Promega) was used to measure the signals 48 h after transfection.

### *In vivo* tumorigenicity and metastasis assays

The Animal Care and Use Committee approved the animal experiment that was performed in accordance with institutional guidelines. BALB/C athymic nude mice were housed under specific pathogen-free conditions. 2×10^6^ CCA cells transfected with miR-16a mimics or inhibitor were injected into hind limbs of mice to generate xenograft tumors. Tumor size was measured and tumor volume was determined by the formula: 0.5 × length × width^2^. For metastasis assay, cells were resuspended in PBS at a concentration of 2×10^7^ cells/ml. A volume of 0.1 mL of suspended QBC939 cells, miR-16 inhibited and overexpressed QBC939 cells were injected into the tail veins. All of the mice were sacrificed after inoculation of 6 weeks, then the metastatic nodes in the lungs were examined by necropsy and counted. The metastatic nude was monitored by the IVIS@ Lumina II system every 5 days.

### Statistical analysis

SPSS v.17.0 and GraphPad Prism software were used for statistical analysis. Analysis of variance, two-sided Fisher's exact test, and student's t test were used to evaluate differences between groups. Data were presented as mean ± SD, and *P* < 0.05 indicated statistically significant.

## SUPPLEMENTARY MATERIALS FIGURES AND TABLES



## Abbreviations

CCA: cholangiocarcinoma
UTR: untranslation region
RT-PCR: real time: polymerase chain reaction
SDS-PAGE: sodium dodecyl sulphate polyacrylamide gel electrophoresis
PVDF: polyvinylidene fluoride
EDU: 5-ethynyl-2′-deoxyuridine
