# Supplementary material for: Suppression of miR-16 promotes tumor growth and metastasis through reversely regulating YAP1 in human cholangiocarcinoma
Source: Oncotarget. 2017 May 12;8(34):56635–50. doi: 10.18632/oncotarget.17832 (PMC5593589; doi:10.18632/oncotarget.17832)
Supplement: Supplementary file 1 [file oncotarget-08-56635-s001.pdf]

# Suppression of miR-16 promotes tumor growth and metastasis through reversely regulating YAP1 in human cholangiocarcinoma

## SUPPLEMENTARY TABLES

**Supplementary Table 1: Candidate miRNAs screened by microarray**

| Candidate miRNAs | FC (abs) | Regulation |
|---|---|---|
| hsa-miR-22_st | 3.441764 | up |
| hsa-miR-93_st | 7.254357 | up |
| hsa-miR-1908_st | 8.018061 | up |
| hsa-miR-663_st | 9.433633 | up |
| hsa-miR-21_st | 14.276008 | up |
| hsa-miR-1228-star_st | 2.517971 | up |
| hsa-miR-720_st | 2.462887 | down |
| hsa-miR-122_st | 4.225059 | down |
| hsa-miR-193b_st | 2.8636 | down |
| hsa-miR-4734_st | 4.86192 | down |
| hsa-miR-16_st | 21.880374 | down |

**Supplementary Table 2: Bioinformatics prediction for potential candidate targets for miR-16**

| miRNA | Targets | miRbase | Target Scan | PicTar | miRNA Target |
|---|---|---|---|---|---|
| **miR-16** | CD80 | √ | √ | √ | √ |
| | KIF21A | √ | √ | | √ |
| | ARL2 | | √ | √ | √ |
| | YAP1 | | √ | √ | √ |
| | MYBL1 | √ | √ | √ | |
| | CCNE1 | √ | √ | √ | |

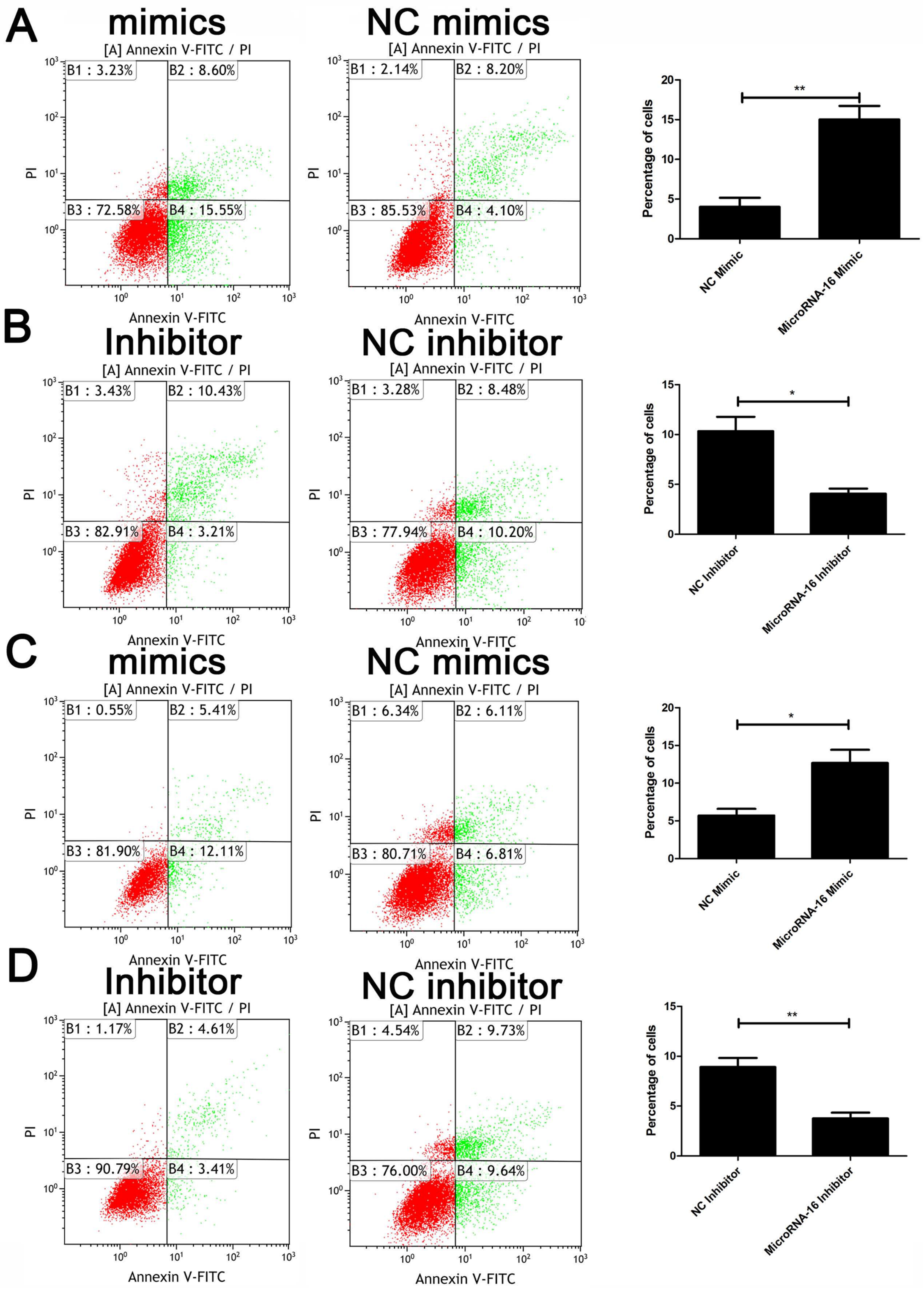


**Supplementary Figure 1A: miR-16 promotes cell apoptosis *in vitro*.** Cells were treated with miR-16 mimics or inhibitor. The apoptosis was detected by flow cytometry. Data are presented as means ± SEM and analyzed with Student t test (**P < 0.01, ***P < 0.001).

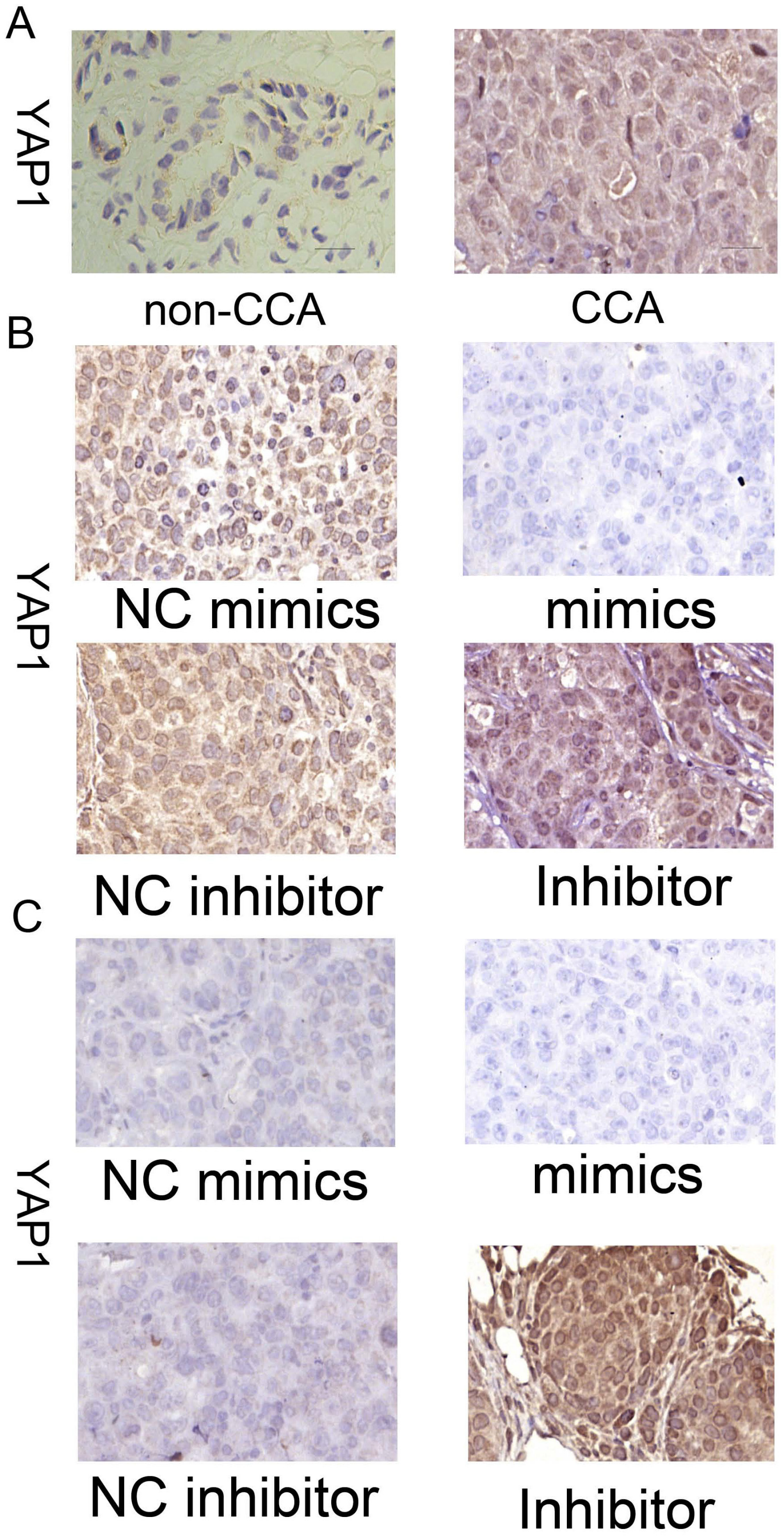


**Supplementary Figure 1B: YAP1 expression in human tissues and tumor tissues *in vivo*** A : The protein expression of YAP1 in human tumor tissues and the adjacent tumor tissues detected by IHC. B : The expression of YAP1 in tumor tissues in vivo with nude murine with tumor subcutaneously implanted by IHC. C : The expression of YAP1 in the lung metastasis nude detected by IHC.
